# Anti‐KIT Barzolvolimab for Chronic Spontaneous Urticaria

**DOI:** 10.1111/all.16598

**Published:** 2025-05-26

**Authors:** Marcus Maurer, Martin Metz, John Anderson, Neetu Talreja, Diane Young, Elizabeth Crowley, Margo Heath‐Chiozzi, Rick Ma, Elsa Paradise, Thomas Hawthorne, Diego Alvarado, Jonathan A. Bernstein

**Affiliations:** ^1^ Institute of Allergology Charité—Universitätsmedizin Berlin Berlin Germany; ^2^ Fraunhofer Institute for Translational Medicine and Pharmacology, Immunology and Allergology Berlin Germany; ^3^ Clinical Research Center of Alabama, AllerVie Health University of Alabama, Birmingham, and VA Medical Center Birmingham Alabama USA; ^4^ The Allergy Group and Treasure Valley Medical Research Boise Idaho USA; ^5^ Celldex Therapeutics Hampton New Jersey USA; ^6^ University of Cincinnati Cincinnati Ohio USA

**Keywords:** anti‐KIT, chronic urticaria, hives, mast cell, monoclonal antibody, stem cell factor

## Abstract

**Background:**

Chronic spontaneous urticaria (CSU) is characterized by mast cell (MC)‐mediated wheals and/or angioedema without identifiable triggers and is driven by MC activation. Barzolvolimab—a monoclonal anti‐KIT antibody—depletes MCs by inhibiting activation of KIT by stem cell factor. We evaluated multiple ascending doses in patients with CSU.

**Methods:**

Phase 1b double‐blind placebo‐controlled trial (NCT04538794) in adults with moderate‐to‐severe (urticaria activity score over 7 days [UAS7] ≥ 16) antihistamine–refractory CSU treated with intravenous barzolvolimab for 12 weeks with a 12‐week follow‐up in four sequentially enrolled cohorts (randomized 4:1 barzolvolimab:placebo): 0.5 mg/kg, Q4W (*n* = 9); 1.5 mg/kg, Q4W (*n* = 8); 3 mg/kg, Q8W (*n* = 9); and 4.5 mg/kg, Q8W (*n* = 9). Primary and secondary objectives were safety and disease activity (UAS7 and urticaria control test [UCT]). Pharmacokinetics and pharmacodynamics were assessed.

**Results:**

Patients had high mean (range) baseline CSU activity, with UAS7 = 29.6 (16.3–42.0) for barzolvolimab‐treated, UAS7 = 35.8 (19.0–42.0) for placebo‐treated, and 44% prior omalizumab use. Multiple doses of barzolvolimab were well tolerated. Hair color change was the commonest adverse event in barzolvolimab‐treated patients. Across barzolvolimab doses, rapid symptom reduction within 1 week was observed and sustained during 12 weeks; 71% of patients achieved a well‐controlled (UAS7 ≤ 6) response and 57% a complete response (UAS7 = 0). Additionally, 77% of barzolvolimab‐treated patients achieved a well‐controlled response (UCT ≥ 12) and 43% a complete response (UCT = 16) by Week 12. The kinetics of disease activity paralleled tryptase suppression, indicative of MC inhibition. Patients with and without prior omalizumab treatment responded similarly.

**Conclusions:**

This study supports barzolvolimab as a promising treatment for CSU.

## Graphical abstract

We compared the efficacy and safety of four doses of barzolvolimab compared to placebo in reducing symptoms of chronic spontaneous urticaria in a randomized, controlled trial. Across barzolvolimab doses, rapid symptom reduction within 1 week was observed and sustained during 12 weeks. The kinetics of disease activity paralleled tryptase suppression, indicative of mast cell inhibition.

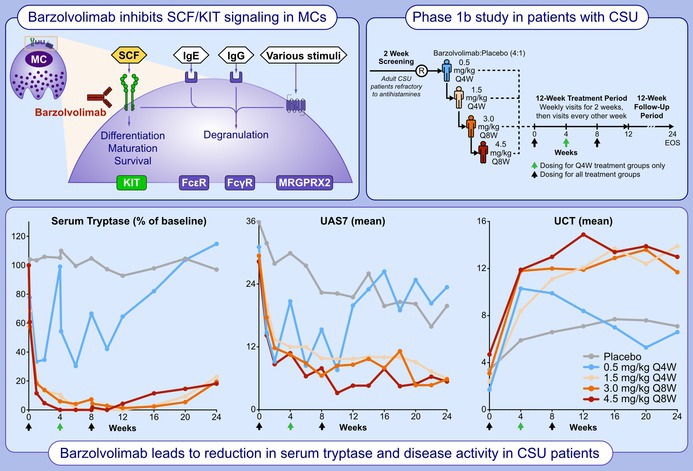

**Abbreviations**
EOS: end of study
FcR: Fc receptor
Ig: immunoglobulin
KIT: receptor tyrosine kinase
MC: mast cell
MRGPRX2: Mas‐related G‐protein coupled receptor member X2
Q4/8W: once every 4/8 weeks
SCF: stem cell factor
UAS7: urticaria activity score over 7 days
UCT: urticaria control test

## 1 Introduction

The signs and symptoms of chronic spontaneous urticaria (CSU) are initiated by mast cell (MC) activation and degranulation [1, 2, 3]. This results in sensory nerve stimulation, vasodilation, extravasation, and the development of itch, wheals, and angioedema [4]. The mechanisms of MC activation in CSU are not fully understood but are known to involve multiple pathways [5]. However, current treatments target specific triggers (e.g., IgE [omalizumab] or MC mediators [antihistamines]) rather than targeting MCs directly. CSU treatment guidelines [1, 3] recommend daily treatment with second‐generation H1 antihistamines at approved doses, with dose escalation up to fourfold the standard dose if CSU symptoms are not controlled and antihistamine side effects are tolerated. Omalizumab, an IgE‐neutralizing monoclonal antibody (mAb), may be added if symptoms are still not controlled. With these treatments, it is estimated that complete or partial disease control is possible for approximately two‐thirds of patients [6]. There remains an unmet need for new therapies to achieve better disease control in patients with CSU, particularly those who do not respond to omalizumab. MCs are the key effector cells in CSU; therefore, treatment targeting MCs directly may be more effective than existing treatments.

Barzolvolimab (CDX‐0159), a first‐in‐class anti‐KIT mAb previously shown to deplete skin MCs and circulating tryptase, has demonstrated improvement in itch and urticarial lesions [7]. MCs require engagement of their KIT receptors by stem cell factor (SCF) for activation, tissue recruitment, and survival [8, 9]. Barzolvolimab, as a potent inhibitor of SCF‐dependent KIT activation in cells, is hypothesized to provide therapeutic benefit in patients with CSU by decreasing the number of MCs and their activity. We report the effect of multiple ascending doses (MAD) of barzolvolimab on safety, pharmacokinetics (PK), pharmacodynamics (PD), immunogenicity, and clinical activity in CSU patients whose disease is inadequately controlled by antihistamines.

## 2 Methods

### 2.1 Trial Design and Oversight

This Phase 1b, randomized, double‐blinded, placebo‐controlled study (NCT04538794) in patients with CSU refractory to antihistamines assessed barzolvolimab safety, PK, and PD compared with placebo. The study included the following periods: 2‐week screening, 12‐week treatment, and 12‐week posttreatment follow‐up (Figure S1).

Doses were selected based on results from the single‐dose healthy‐volunteer study [10], in which 0.3–9 mg/kg barzolvolimab was well tolerated and demonstrated dose‐dependent suppression of serum tryptase (Supporting Information Methods). Given the sustained tryptase suppression and prolonged serum half‐life observed in the single‐dose study, two dose schedules were evaluated: once every 4 weeks (Q4W) and every 8 weeks (Q8W).

This trial was designed and sponsored by Celldex Therapeutics. The institutional review board at each participating center approved the protocol. Patients provided written informed consent before any assessment was performed. Data were collected by the trial investigators according to Good Clinical Practice guidelines and were analyzed by the sponsor. The first draft of the manuscript was written by a medical writer paid by the sponsor, with critical input and approval from all authors. All the authors critically reviewed each manuscript draft, provided substantial input on the content, and made the decision to submit the manuscript for publication. The authors vouch for the accuracy and completeness of the data and the fidelity of the trial to the protocol.

### 2.2 Patients

Eligible patients were aged 18–75 years and had CSU refractory to H1 antihistamine at approved or increased doses alone or in combination with H2 antihistamine and/or leukotriene receptor antagonists (LTRA). Key inclusion criteria were moderate‐to‐severe CSU, defined as a weekly urticaria activity score (UAS7) of ≥ 16 (disease activity categories: 0, symptom free; 1–6, well‐controlled; 7–15, mild; 16–27, moderate; and 28–42, severe urticaria [11]); a weekly Hives Severity Score (HSS7) ≥ 8 (range: 0–21; higher values indicate greater severity) during the 7 days prior to randomization (Day 1); an in‐clinic UAS ≥ 4 on ≥ 1 screening visit days; antihistamine for treatment of CSU for at least 3 consecutive days immediately prior to the initial screening visit and were expected to use the antihistamines throughout the study period beginning at screening; CSU diagnosis for ≥ 6 months; and presence of itch and hives for ≥ 6 consecutive weeks at any time prior to enrollment despite concurrent use of antihistamine. Patients met defined hematology and chemistry criteria; had no other comorbidities that would introduce additional risk factors or interfere with study procedures; had no history of anaphylaxis; and had no known HIV, hepatitis B, or hepatitis C infection.

Key exclusion criteria were receipt of prior biologic therapy (e.g., omalizumab, dupilumab, and ligelizumab) within the past 3 months or prior treatment with barzolvolimab; treatment with immunosuppressives within 4 weeks/5 half‐lives; treatment with doxepin (oral) within 4 weeks prior to study treatment; and any other skin disease associated with chronic itching that might confound study evaluations and results.

### 2.3 Trial Procedures

Patients were randomly assigned in a 4:1 ratio to receive a 60‐min intravenous infusion of either barzolvolimab or placebo (saline) Q4W or Q8W according to their assigned cohort (1: 0.5 mg/kg Q4W; 2: 1.5 mg/kg Q4W; 3: 3.0 mg/kg Q8W; and 4: 4.5 mg/kg Q8W) over 12 weeks as add‐on treatment to H1 antihistamine either alone or in combination with H2 antihistamine and/or LTRA (administered prior to study drug infusion). Rescue treatments were allowed up to maximum allowable doses: diphenhydramine 100 mg 4× daily (QID), hydroxyzine 50 mg QID, or H2 antihistamine 4× the recommended dose.

### 2.4 End‐Point Measures

The main objective of the trial was to evaluate the barzolvolimab MAD safety profile. Safety was assessed by the incidence and severity of treatment‐emergent adverse events (TEAEs) and serious AEs (SAEs), dose‐limiting toxicities, clinical laboratory tests, vital sign measurements, 12‐lead electrocardiograms, and physical examinations. The secondary objectives were to characterize the barzolvolimab MAD PK profile; PD effect (UAS7, HSS7, itch severity score [ISS7], and urticaria control test [UCT]); tryptase and SCF levels; and immunogenicity. Supporting Information Methods has details on secondary and exploratory objectives.

### 2.5 Statistical Analyses

Results were analyzed by dose and overall for patients who received barzolvolimab. The placebo group was pooled from each cohort. Baseline was defined as the last available measurement prior to study treatment administration. Change from baseline was calculated as the difference between postbaseline and baseline values.

Safety was assessed separately by treatment group and as pooled active treatment. Analyses included the number and percentage of patients experiencing AEs, summarized by severity and relationship to study drug. Laboratory parameters were presented as summaries of actual values and changes from baseline.

PK parameters were calculated using noncompartmental methods and summarized using descriptive statistics to compare exposure in single and multiple dose settings. The impact of antidrug antibodies (ADA) on drug levels and safety was evaluated by comparing the area under the concentration–time curve (AUC) and the incidence of AEs for ADA‐positive versus ADA‐negative patients in each treatment group, respectively. Summary statistics were descriptive and presented to evaluate PD changes. SAS Version 9.4 was used for statistical analysis.

## 3 Results

### 3.1 Participants

A total of 74 patients were screened, of whom 45 were randomly assigned. Overall, 42 patients (93.3%) completed treatment (Figure S2). Three patients did not complete the treatment period: one in the barzolvolimab 4.5‐mg/kg group because of low white blood count (WBC), and two in the placebo group who sought alternate treatment for CSU because of symptom severity. The analysis population was the safety population, defined as all patients who received at least one dose of study treatment. There were no notable differences between the barzolvolimab and placebo groups in the demographic or clinical characteristics at baseline (Table 1). Most patients had severe disease per UAS7. A total of 44% of patients had prior use of biologics. The percentage of patients with a history of angioedema was similar between placebo (50%) and across the barzolvolimab groups (60%), with mean baseline angioedema activity score over 7 days (AAS7) of 26.6 and 26.0, respectively.

**TABLE 1:** Demographics and baseline characteristics.

Characteristics	Barzolvolimab 0.5 mg/kg Q4W (*N* = 9)	Barzolvolimab 1.5 mg/kg Q4W (*N* = 8)	Barzolvolimab 3.0 mg/kg Q8W (*N* = 9)	Barzolvolimab 4.5 mg/kg Q8W (*N* = 9)	All barzolvolimab (*N* = 35)	Pooled placebo (*N* = 10)
Age, years	43.8 (21–73)	53.3 (29–75)	49.4 (26–65)	51.1 (29–68)	49.3 (21–75)	49.8 (18–70)
Gender, female, *n* (%)	6 (67)	7 (88)	6 (67)	9 (100)	28 (80)	7 (70)
BMI kg/m^2^	31.1 (26.0–36.0)	37.8 (28.6–58.9)	29.4 (22.3–36.9)	27.1 (21.5–34.4)	31.2 (21.5–58.9)	31.8 (16.4–55.2)
CSU duration, years	7.5 (0.6–41.1)	17.1 (2.6–61.3)	4.8 (0.6–21.3)	10.4 (1.0–35.4)	9.7 (0.6–61.3)	5.6 (1.4–13.1)
Prior H2‐antihistamine treatment *n* (%)	6 (67)	4 (50)	4 (44)	6 (67)	20 (57)	4 (40)
Prior leukotriene receptor antagonist treatment, yes, *n* (%)	3 (33)	2 (25)	0 (0)	3 (33)	8 (23)	2 (20)
Prior omalizumab^a^ *n* (%)	4 (44)	3 (38)	4 (44)	2 (22)	13 (37)	6 (60)
UAS7	31.1 (20.0–39.0)	29.5 (20.0–40.6)	29.4 (16.3–42.0)	28.3 (22.0–38.0)	29.6 (16.3–42.0)	35.8 (19.0–42.0)
AAS7 at baseline^b^ *n* (%)	5 (56)	6 (75)	7 (78)	8 (89)	26 (74)	5 (50)
AAS7^b^ mean (range)	29.0 (4.0–59.5)	43.3 (1.0–79.0)	29.8 (3.0–49.0)	33.7 (6.0–80.0)	34 (1.0–80.0)	53.2 (3.5–80.5)
UCT	1.7 (0–4)	2.4 (1–8)	3.1 (0–7)	4.7 (1–12)	3.0 (0–12)	3.4 (0–11)
Tryptase, ng/mL	5.0 (2.0–10.3)	6.3 (2.8–15.1)	8.6 (3.3–28.8)	5.5 (2.3–10.2)	6.2 (2.0–28.8)	5.3 (3.2–7.5)

*Note:* Mean and range are presented unless otherwise indicated.

Abbreviations: AAS7, angioedema activity score over 7 days; BMI, body mass index; CSU, chronic spontaneous urticaria; Q4W, every 4 weeks; Q8W, every 8 weeks; UAS7, urticaria activity score over 7 days; UCT, urticaria control test.

^a^ The majority had an inadequate response to omalizumab.

^b^ Only patients who were positive for AAS7 at baseline.

### 3.2 Safety

Overall, 30 barzolvolimab‐treated patients (86%) and 6 placebo‐treated patients (60%) experienced a TEAE. In the 1.5‐mg/kg group, two barzolvolimab‐treated patients (6%) experienced infusion‐related TEAEs. One patient (3%) in the 3.0‐mg/kg group experienced a TEAE that led to drug interruption. No patients experienced a dose‐limiting toxicity or any TEAE that led to treatment discontinuation. Hair color change was the most common event, experienced by nine barzolvolimab‐treated patients (26%; Table 2); hair color changes occurred in different locations: scalp, beard, and body hair; and seven patients had this TEAE resolve by the end of the study. Neutropenia, headache, and COVID‐19 infection were the second‐most common TEAEs, with five barzolvolimab‐treated patients (14%) experiencing each of these. The TEAEs of nasopharyngitis and urinary tract infection (UTI) were experienced by four barzolvolimab‐treated patients (11%). Fatigue, nasopharyngitis, and headache were reported in the placebo group. A total of 40% of barzolvolimab‐treated patients and 20% of placebo‐treated patients experienced an infection AE. The infections that were reported by > 10% of barzolvolimab‐treated patients were COVID‐19 (14%), nasopharyngitis (11%), and UTI (11%), and were not correlated with neutrophil decreases.

**TABLE 2:** TEAEs reported by system organ class and preferred term in ≥ 10% barzolvolimab‐treated patients at 24 weeks.

Characteristics	Barzolvolimab 0.5 mg/kg Q4W (*N* = 9)	Barzolvolimab 1.5 mg/kg Q4W (*N* = 8)	Barzolvolimab 3.0 mg/kg Q8W (*N* = 9)	Barzolvolimab 4.5 mg/kg Q8W (*N* = 9)	All barzolvolimab (*N* = 35)	Pooled placebo (*N* = 10)
Any TEAE^a^	8 (89)	7 (88)	9 (100)	6 (67)	30 (86)	6 (60)
Blood and lymphatic system disorders	2 (22)	3 (38)	4 (44)	0 (0)	9 (26)	0 (0)
Neutropenia	2 (22)	2 (25)	1 (11)	0 (0)	5 (14)	0 (0)
Leukopenia	1 (11)	1 (13)	1 (11)	0 (0)	3 (9)	0 (0)
Gastrointestinal disorders	1 (11)	2 (25)	2 (22)	2 (22)	7 (20)	1 (10)
Diarrhea	1 (11)	0 (0)	1 (11)	1 (11)	3 (9)	0 (0)
General disorders and administration site conditions	1 (11)	1 (13)	1 (11)	1 (11)	4 (11)	1 (10)
Fatigue	1 (11)	1 (13)	0 (0)	1 (11)	3 (9)	1 (10)
Infections and infestations	1 (11)	4 (50)	5 (56)	4 (44)	14 (40)	2 (20)
COVID‐19	0 (0)	1 (13)	2 (22)	2 (22)	5 (14)	0 (0)
Nasopharyngitis	0 (0)	1 (13)	2 (22)	1 (11)	4 (11)	1 (10)
Urinary tract infection	1 (11)	2 (25)	1 (11)	0 (0)	4 (11)	0 (0)
Muscular and connective tissue disorders	2 (22)	3 (38)	3 (33)	1 (11)	9 (26)	0 (0)
Arthralgia	2 (22)	0 (0)	0 (0)	1 (11)	3 (9)	0 (0)
Back pain	0 (0)	1 (13)	2 (22)	0 (0)	3 (9)	0 (0)
Nervous system disorders	4 (44)	0 (0)	4 (44)	2 (22)	10 (29)	2 (20)
Headache	2 (22)	0 (0)	2 (22)	1 (11)	5 (14)	2 (20)
Skin and subcutaneous tissue disorders	1 (11)	2 (25)	5 (56)	6 (67)	14 (40)	0 (0)
Hair color changes	0 (0)	1 (13)	3 (33)	5 (56)	9 (26)	0 (0)

Abbreviations: Q4W, every 4 weeks; Q8W, every 8 weeks; TEAEs, treatment‐emergent adverse events.

^a^ Taste changes, as noted in prior barzolvolimab studies, were reported by three patients (< 10%).

Most AEs were mild or moderate in severity and resolved while on study. One SAE of Grade 4 salmonella gastroenteritis in a 59‐year‐old Black woman (1.5‐mg/kg group) was assessed by the investigator as unrelated to study treatment; she was treated with Levaquin, was discharged, and completed the study as planned.

Decreases in leukocytes, absolute neutrophil count, hemoglobin, and platelets were observed in patients after the first barzolvolimab dose when compared with placebo; no additional decreases were observed after subsequent doses (Figure S3). The majority of shifts were to Grades 1 and 2. A patient who received 4.5‐mg/kg barzolvolimab experienced a Grade 3 decrease in platelets; there was one patient also in the 4.5‐mg/kg group who experienced a Grade 2 decrease in WBC and did not receive the second dose per protocol; and three patients experienced Grade 3 decreased neutrophils (one in the 1.5‐mg/kg group and two in the 4.5‐mg/kg group); these all resolved during the study. There was no correlation of decreases with dose level. There were no trends of neutropenia correlating with increased infections.

Fifty‐seven percent (20/35) of patients were ADA positive, and the proportion of patients who experienced drug‐related AEs was similar in ADA‐positive and ADA‐negative patients.

### 3.3 Secondary End Points

**FIGURE 1:**
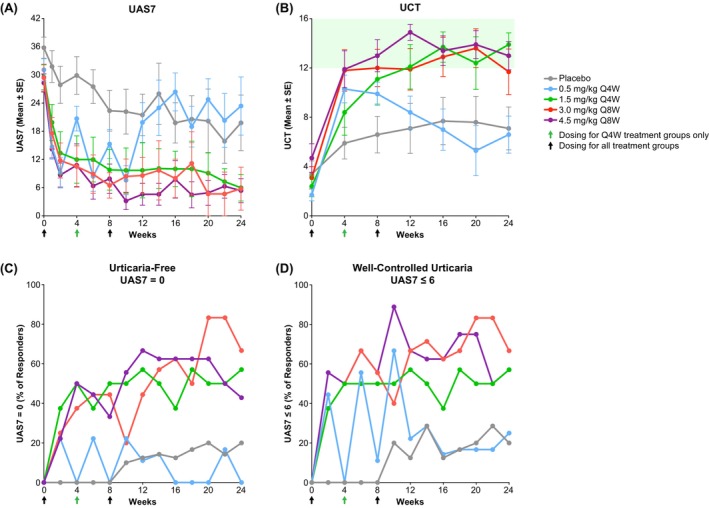
Change from baseline in UAS7 and UCT and percentage of responders for UAS7 = 0 and UAS7 ≤ 6 according to barzolvolimab dose. (A) UAS7 mean ± SE shown from baseline to Week 24. UAS7 describes urticaria activity, with lower scores representing low urticaria activity and higher scores representing high urticaria activity. (B) UCT mean ± SE shown from baseline to Week 24. Higher scores represent greater urticaria control, with a score of ≥ 12 indicating well‐controlled disease (shaded light green). (C) Percentage of patients who were urticaria free (UAS7 = 0) from baseline to Week 24. (D) Percentage of patients who were well controlled (UAS7 ≤ 6) from baseline to Week 24. Abbreviations: Q4W, every 4 weeks; SE, standard error; UAS7, urticaria activity score over 7 days; UCT, urticaria control test.

Urticaria symptoms measured by change from baseline in UAS7 improved by Week 1 (Figure 1A) for all barzolvolimab dose levels compared with placebo. Sustained improvement of UAS7 scores through Week 24 was seen for the groups ≥ 1.5 mg/kg. HSS7 and ISS7, the two components of UAS7 score, showed comparable improvement in each urticaria symptom measurement by Week 1 and sustained improvement of scores through Week 24 for the ≥ 1.5‐mg/kg groups (Figure S4). Sustained improvement was also observed by change from baseline in UCT (Figure 1B).

UAS7 response to barzolvolimab was greater than placebo: 71.4% of barzolvolimab‐treated patients had a well‐controlled response (UAS7 ≤ 6) by Week 12, compared with 30.0% of placebo‐treated patients (Figure 1C), and 57.1% of barzolvolimab‐treated patients had a complete response (UAS7 = 0) by Week 12 compared with 20% of placebo‐treated patients (Figure 1D). Overall, 77.1% of barzolvolimab‐treated patients achieved a well‐controlled response (UCT ≥ 12), and 42.9% of the patients who received barzolvolimab achieved a complete response (UCT = 16) by Week 12.

**FIGURE 2:**
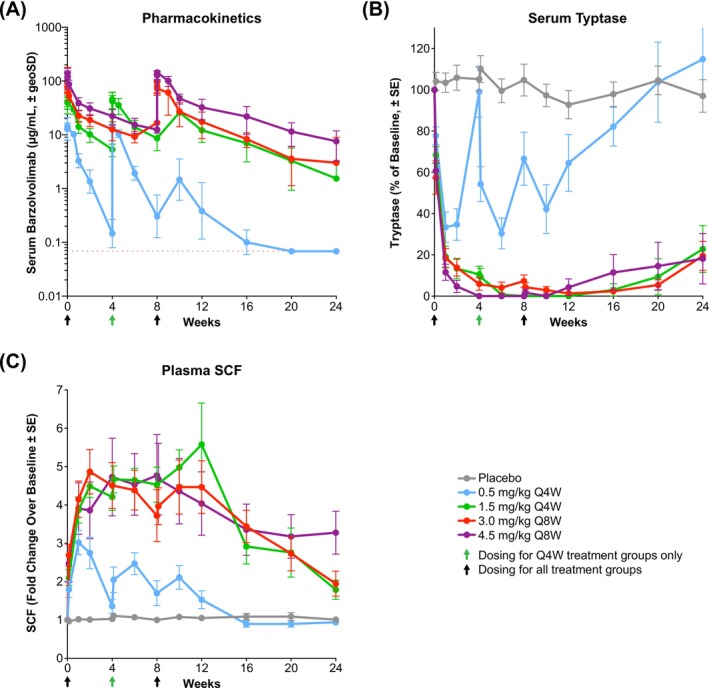
Change from baseline in serum barzolvolimab concentrations, serum tryptase percentage of baseline, and plasma SCF fold change over baseline according to barzolvolimab dose. (A) Serum barzolvolimab concentrations mean ± GeoSD, from baseline to Week 24, dotted red line indicates lower limit of quantitation 0.07 ug/mL. (B) Serum tryptase mean percentage of baseline ± SE. (C) Plasma SCF mean fold change over baseline ± SE. Abbreviations: GeoSD, geometric standard deviation; SCF, stem cell factor; SE, standard error.

Multiple doses of barzolvolimab resulted in prolonged exposure through 24 weeks in the ≥ 1.5‐‍mg/kg dose levels (Figure 2A). Maximum serum concentration (C_max_) and AUC increased in a linear fashion with increasing dose (Table S1). Increased clearance and lower volumes of distribution were observed in patients who received lower doses. Treatment‐emergent ADA were observed in 57% of barzolvolimab‐treated patients, without apparent impact on exposure. Serum tryptase, a specific marker for MCs, was suppressed at all barzolvolimab doses, and reduction below assay detection levels was generally observed at doses ≥ 1.5 mg/kg (Figure 2B). Maximal tryptase suppression and clinical activity were reached at doses ≥ 1.5 mg/kg, which are consistent with target saturation. Plasma SCF increases mirrored the tryptase suppression kinetics, and both correlated well with clinical activity (Figure 2C).

**FIGURE 3:**
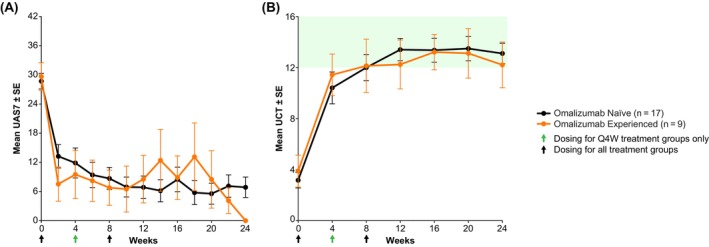
Change from baseline in UAS7 and UCT in barzolvolimab‐treated patients according to omalizumab naivety or experience. (A) UAS7 mean ± SE in omalizumab‐naive and ‐experienced patients from baseline to Week 24. UAS7 calculated over 7 consecutive days describes urticaria activity, with lower scores representing low urticaria activity and higher scores representing high urticaria activity. (B) UCT mean ± SE in omalizumab‐naive and ‐experienced patients from baseline to Week 24. Higher scores represent greater urticaria control, with a score of ≥ 12 indicating well‐controlled disease. Pooled data from saturating doses (1.5, 3.0, and 4.5 mg/kg). Abbreviations: Q4W, every 4 weeks; SE, standard error; UAS7, urticaria activity score over 7 days; UCT, urticaria control test.

A subgroup analysis of patients who had previously received omalizumab had a similar response profile (UAS7 and UCT) to those who were naive to omalizumab (Figure 3); the majority of experienced patients had discontinued prior omalizumab because of a lack of symptom control. Doses ≥ 1.5 mg/kg were pooled in barzolvolimab‐treated patients, and a complete response (UAS7 = 0) and well‐controlled response (UAS7 ≤ 6) by Week 12 were observed in 53.8% and 84.6% of barzolvolimab‐treated patients with prior omalizumab treatment, respectively, which is similar to the overall group.

### 3.4 Exploratory End Points

The AAS7 results demonstrated rapid, durable symptom improvement in angioedema in the barzolvolimab groups (Figure S5). Decreases in the Dermatology Life Quality Index (DLQI) mean scores for the ≥ 1.5‐mg/kg barzolvolimab groups were sustained through Week 24 (baseline score = 11.3 in pooled barzolvolimab group vs. Week 24 mean scores = 1.3 [1.5 mg/kg], 3.2 [3.0 mg/kg], and 2.0 [4.5 mg/kg]; Figure S6A). Physician Global Assessment (PhysGA) showed decreases starting at Week 1 (mean baseline score for pooled barzolvolimab group = 2.2 vs. 0.6 [Week 1]), which were sustained through Week 24 (Week 24 = 0.8 [1.5 mg/kg], 0.4 [3.0 mg/kg], and 0.6 [4.5 mg/kg]; Figure S6B).

## 4 Discussion

For patients with severe CSU, symptom control can be difficult to attain with available therapies. Multiple doses of barzolvolimab up to 4.5 mg/kg were well tolerated in patients with antihistamine–refractory moderate‐to‐severe CSU, and the safety profile of barzolvolimab through 24 weeks was similar to single‐dose studies [10]. Hematologic parameters decreased after the first dose and showed no pattern of further decreases with multiple doses. The efficacy assessments showed rapid and sustained improvement of urticaria symptoms from baseline to postdose timepoints with barzolvolimab doses ≥ 1.5 mg/kg, regardless of prior omalizumab treatment, consistent with the central role MCs play in CSU. These results support the anti‐KIT mechanism of action for treating CSU.

The observed AEs in barzolvolimab‐treated patients reflect KIT‐mediated toxicities and were reversible, consistent with the expected effects of KIT inhibition. The most common AE related to treatment with barzolvolimab was mild hair color changes, followed by mild‐to‐moderate neutropenia, which was not associated with infections. Decreases in hematologic parameters were consistent with observations in healthy volunteers [10] and patients with chronic inducible urticaria [7] who received a single barzolvolimab dose. Emerging data from an ongoing Phase 2 study with barzolvolimab administered subcutaneously in CSU (NCT05368285) supports the low risk associated with hematologic parameter decreases in this population. These Phase 1 data indicate that this first‐in‐class anti‐KIT mAb was well tolerated.

Sustained clinical response with barzolvolimab mirrored the changes in tryptase and SCF, suggesting that depleting MCs is effective in improving CSU symptoms. A total of 71% of barzolvolimab‐treated patients across doses achieved a well‐controlled (UAS7 ≤ 6) response, and 57% of the patients achieved a complete response (UAS7 = 0) during the first 12 weeks (compared with 36% of participants treated with omalizumab 300 mg Q4W in registrational trials at 12 weeks [12]); this was sustained through Week 24. The UAS7 results were supported by data from its components, HSS7 and ISS7, as well as the UCT score results. Patients had similar symptom improvement irrespective of prior omalizumab use, suggesting that barzolvolimab can help improve urticaria symptoms regardless of whether IgE is the underlying trigger. Barzolvolimab treatment resulted in rapid, durable symptom improvement in angioedema. The increased symptom control observed with barzolvolimab was reflected in the quality of life measurements, which showed profound improvement between Weeks 1 and 4 that was sustained through Week 24.

The kinetics of clinical response were rapid and strongly associated with changes in tryptase and SCF levels, consistent with fast‐target engagement leading to MC inactivation and subsequent depletion. Target saturation was observed with a durable clinical response and tryptase suppression with doses ≥ 1.5 mg/kg. As tryptase levels were suppressed, improvement in UAS7 was observed within 1 week, and > 50% of patients at doses ≥ 1.5 mg/kg remained urticaria free at Week 24, 16 weeks after their last dose. The lowest dose (0.5 mg/kg) showed transient improvements in clinical activity that correlated with partial tryptase suppression, SCF increases, and subsequent recovery prior to the following dose. All KIT‐dependent functions, including tissue MCs, hematological parameters, and hair color changes, are expected to recover once KIT signaling resumes following drug clearance, as has been observed in prior studies with barzolvolimab.

Barzolvolimab levels accumulated through multiple dosing cycles at all dose levels, and exposure by C_max_ and AUC increased linearly with dose. Immunogenicity, as defined by the occurrence of treatment‐emergent ADA response, was observed in 57% of patients, but no impact on PK was apparent. Antibarzolvolimab antibodies did not appear to impact the safety parameters in this study.

In patients with moderate‐to‐severe CSU, barzolvolimab was well tolerated through Week 24 and resulted in rapid and sustained symptom control after multiple doses ≥ 1.5 mg/kg. This study was limited by the small sample size and variation in drugs used for background urticaria treatment; however, patients had to be on a stable regimen prior to barzolvolimab administration. Larger trials are ongoing to establish barzolvolimab clinical safety and efficacy in patients with CSU and are planned in patients with chronic inducible urticaria, where MCs also play a central role. This study initially characterizes barzolvolimab as a promising novel treatment in antihistamine–refractory CSU.

## Author Contributions

Marcus Maurer, Martin Metz, Jonathan A. Bernstein, Diane Young, Margo Heath‐Chiozzi, Thomas Hawthorne, Diego Alvarado, Rick Ma, and Elizabeth Crowley designed the study. Margo Heath‐Chiozzi, Elizabeth Crowley, Rick Ma, Elsa Paradise, Thomas Hawthorne, Diego Alvarado, and Diane Young coordinated the data collection from the investigators and led the analysis of the data with input from Marcus Maurer, John Anderson, Neetu Talreja, and Jonathan A. Bernstein were involved in the acquisition of data and provided input on CSU and patient diversity. All authors reviewed and provided final approval of the manuscript (Marcus Maurer provided scientific input).

## Conflicts of Interest

Diego Alvarado, Diane Young, Elizabeth Crowley, Elsa Paradise, Rick Ma, Thomas Hawthorne, and Margo Heath‐Chiozzi are full‐time employees of Celldex Therapeutics and may hold stock and/or stock options. Martin Metz reports being a speaker and/or advisor for AbbVie, Advanz, ALK Abello, Allegria, Almirall, Amgen, AstraZeneca, Argenx, Astria, Attovia, Berlin‐Chemie, Celldex, Celltrion, DeepApple, Escient, Galderma, GSK, Incyte, Jasper, Lilly, Novartis, Pfizer, Regeneron, Sanofi, Santa Ana Bio, Septerna, Teva, ThirdHarmonic Bio, and Vifor. John Anderson is a speaker bureau member for CSL Behring, Pharming, BioCryst, Takeda, AstraZeneca, and GSK; has received consulting fees from CSL Behring, Pharming, BioCryst, Pharvaris, Ionis, Takeda, and Novartis; and is a clinical trial investigator for BioCryst, CSL Behring, Pharvaris, Kalvista, Biomarin, Astria, Takeda, Novartis, and Celldex. Neetu Talreja has been an investigator for 9Meters, AbbVie, Allakos, Amgen, Anaptybio Inc., Arena, ARS, AstraZeneca, Biohaven, Braintree, Celldex, Eli Lilly, Escient, GlaxoSmithKline, Gossamer, Incyte, Janssen, Knopp, Nerre, Novartis, Pearl Therapeutics, Pfizer, Phathom, Regeneron, Sanofi, Teva, and Upstream. She has also sat on advisory board(s) and been a medical consultant for Celldex, Novartis, and Regeneron. Jonathan A. Bernstein has been a PI, consultant, and speaker for Novartis, Genentech, AstraZeneca, Sanofi, Regeneron, BioCryst, CSL Behring, Takeda/Shire, Pharming, and GSK; PI and consultant for Celldex, Cogent, Escient, Jasper, Amgen, Roche, Ionis, Kalvista, Allakos, Biomarin, and Blueprint Medicine; PI for Allergy Therapeutics, Telios, Intellia, Aretrea, Pharvaris, Astria; PI for Teva; and consultant for Incyte, Astria, ONO, Cycle, Escient, Pharvaris, and TLL.

## Supporting information


Appendix S1.

## Data Availability

The data that support the findings of this study are available on request from the corresponding author. The data are not publicly available due to privacy or ethical restrictions.
