# Supplementary material for: Anti‐KIT Barzolvolimab for Chronic Spontaneous Urticaria
Source: Allergy. 2025 May 26;80(8):2178–86. doi: 10.1111/all.16598 (PMC12368744; doi:10.1111/all.16598)
Supplement: Supplementary file 1 — Appendix S1. [file ALL-80-2178-s001.docx]

# **Supplemental Methods**

**STUDY DESIGN**

The first patient visited the clinic September 24, 2020, and the last patient visited January 17, 2023.

Doses were selected based on simulations of PK and tryptase response using compartmental modeling and an indirect response PK/PD model derived from Phase 1 healthy-volunteer data.^10^ Comparison of Phase 1 safety data with exposure and tryptase response led to the selected doses. Dosing at 0.5 mg/kg Q4W was predicted to allow partial recovery of tryptase levels before subsequent doses, while 1.5 mg/kg Q4W, 3.0 mg/kg Q8W, and 4.5 mg/kg Q8W were predicted to fully suppress tryptase levels and could predict long-term effects in clinical parameters.

Cohorts 1 and 2 received 3 doses, and Cohorts 3 and 4 received 2 doses. Each cohort was designed with 10 patients, with 8 patients receiving barzolvolimab and 2 patients receiving placebo, for a total of 40 patients (32 barzolvolimab and 8 placebo); 45 patients were enrolled. Dose escalation occurred after all patients within a cohort initiated treatment and >7 patients had received >2 doses and had been followed for 2 weeks after the second dose, with not more than 2 dose-limiting toxicities.

Blinding was managed through the unblinded pharmacists/unblinded staff at the clinical sites, who were provided the treatment allocations by the unblinded staff at IQVIA, when a patient was eligible for randomization. After screening, patients were randomly assigned to a treatment group within the cohort at a 4:1 ratio of active treatment or placebo using a manual randomization method. The block sizes were 5, and completion of a block initiated the assignment of the next random block until all patients were enrolled.

The demographic and clinical characteristics of the patients and the disease activity were elicited or measured at baseline.

**END-POINT MEASUREMENTS**

Exploratory objectives included assessment of the impact of barzolvolimab on quality of life through the Dermatology Life Quality Index (DLQI), angioedema through the Angioedema Activity score (AAS), urticaria symptoms through the Physician Global Assessment (PhysGA), and the percentage of patients who responded to barzolvolimab.

**Safety**

The severity of AEs was evaluated according to the National Institutes of Health Common Terminology Criteria for AE (CTCAE) version 5.0 for infusion reactions. For all other events, severity was assessed using the Toxicity Grading Scale for Healthy Adult and Adolescent Volunteers Enrolled in Preventative Clinical Trials (September 2007).

Other evaluations to measure the safety and tolerability of study treatment included clinical laboratory tests, vital sign measurements, physical examination, electrocardiograms, and pregnancy tests. Because of the theoretical risk associated with depleting MCs and the neutropenia observed with barzolvolimab treatment, infections were reviewed.

**Pharmacokinetics**

Concentrations of barzolvolimab were determined using GLP-compliant enzyme-linked immunoabsorbent assay methods. Blood samples were collected for the measurement of barzolvolimab prior to and after each infusion at each dose level for determination of exposure. Because patients received 3 infusions in Cohorts 1 and 2 and 2 infusions in Cohorts 3 and 4, collection was based on dosing schedules. In Cohorts 1 and 2, samples were collected prior to dosing on study Days 1, 29 and 57, within 15 minutes after completion of infusion, and at 1 hour (± 15 minutes) and 4 hours after the completion of the infusion. Additional samples were collected on study Days 2, 8, 15, 43, 58, 71, 85, 113, 141, and 169. In Cohorts 3 and 4, pre-infusion samples were collected on study Days 1 and 57, at end of infusion, and 1 hour after end of infusion. PK samples were also collected on study Days 2, 8, 15, 29, 43, 58, 71, 85, 113, 141, and 169. Blood was processed to serum at the clinical facility and shipped on dry ice to the Celldex Therapeutics testing facility for accessioning and analysis. Serum was analyzed with a sandwich immunoassay using a validated method with electrochemiluminescent detection on the MesoScale Discovery (MSD) platform with a lower limit of quantitation of 0.07 µg/mL.

Noncompartmental analysis of serum concentrations of barzolvolimab was performed for individual patients using Phoenix® WinNonlin® version 8.4 (Certara USA, Inc., Princeton, NJ) and a plasma model with intravenous infusion. Linear trapezoidal with linear interpolation was used to estimate area under the curve. At a minimum, the final 3 concentrations were used to estimate the terminal phase parameters with uniform weighting, and additional points were added if doing so improved the goodness of fit. Descriptive statistics were used to characterize the relationship of drug dose to exposure.

**Immunogenicity**

Samples were collected pre-dose at Visit 3 and at each visit through Visit 15. CDX-0159 anti-drug antibodies were detected in patient serum samples using an electrochemiluminescent immunoassay with solid phase extraction and acid dissociation. A tiered approach using a screening assay with a 5% false positive rate and a confirmatory assay with a 1% false positive rate were designed to determine the incidence and specificity of any positive samples.

**Tryptase and Stem Cell Factor Analysis**

Serum samples for tryptase and plasma samples for stem cell factor (SCF) were collected for analyses at pre-dose at Visit 3 and at each visit through Visit 15 to measure the change from baseline.

Tryptase was measured at Mayo Clinic Laboratories using the ImmunoCAP® tryptase assay, a fluorescence immunoassay that detects all forms of human alpha- and beta-tryptase (ThermoScientific). The normal range of the assay is 1.0 to 11.5 ng/mL.

SCF was quantified using an electrochemiluminescent method on the MSD platform. SCF standards, controls, and test samples were captured on an MSD standard 96-well plate coated with anti-human SCF antibody MAB655 (R&D Systems). Bound SCF was detected with biotinylated anti-human SCF detection antibody BAF255 (R&D Systems), followed by Sulfo-Tag Streptavidin. The standard curve fitting was performed using the 4-parameter logistic model using a weighting function (1/y^2^), and SCF concentrations in test samples were interpolated from the standard curve. Curve fitting and interpolation were performed using the MSD DISCOVERY WORKBENCH Version 4.0 software.

# **Supplemental Figures**

**Supplemental Figure 1.** **Study Design.** Note that, during the post-treatment follow-up, follow-up consisted of 3 visits every 4 weeks, with the final visit occurring 12 weeks after the end of treatment visit. Abbreviations: CSU, chronic spontaneous urticaria; Q4W, every 4 weeks; Q8W, every 8 weeks.


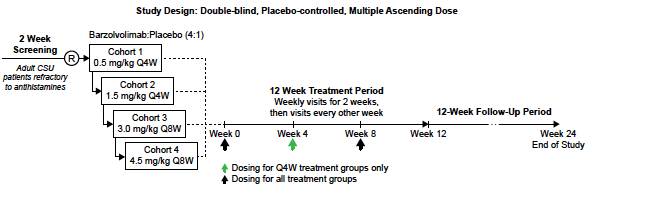


**Supplemental Figure 2.** **CONSORT Diagram.** Flow chart describing the number of patients assessed for eligibility, excluded, randomized, treated, lost to follow-up, and analyzed during the course of the study.

**Supplemental Figure 3. Changes from Baseline in Leukocyte Count, Neutrophil Count, Hemoglobin, and Platelet Count According to Barzolvolimab Dose.** A) Leukocyte count, B) neutrophil count, C) hemoglobin, and D) platelet count. For all panels, the mean score ± SE is shown from baseline to Week 24 in each dose cohort. Abbreviations: SE, standard error; Q4W, every 4 weeks; Q8W, every 8 weeks.


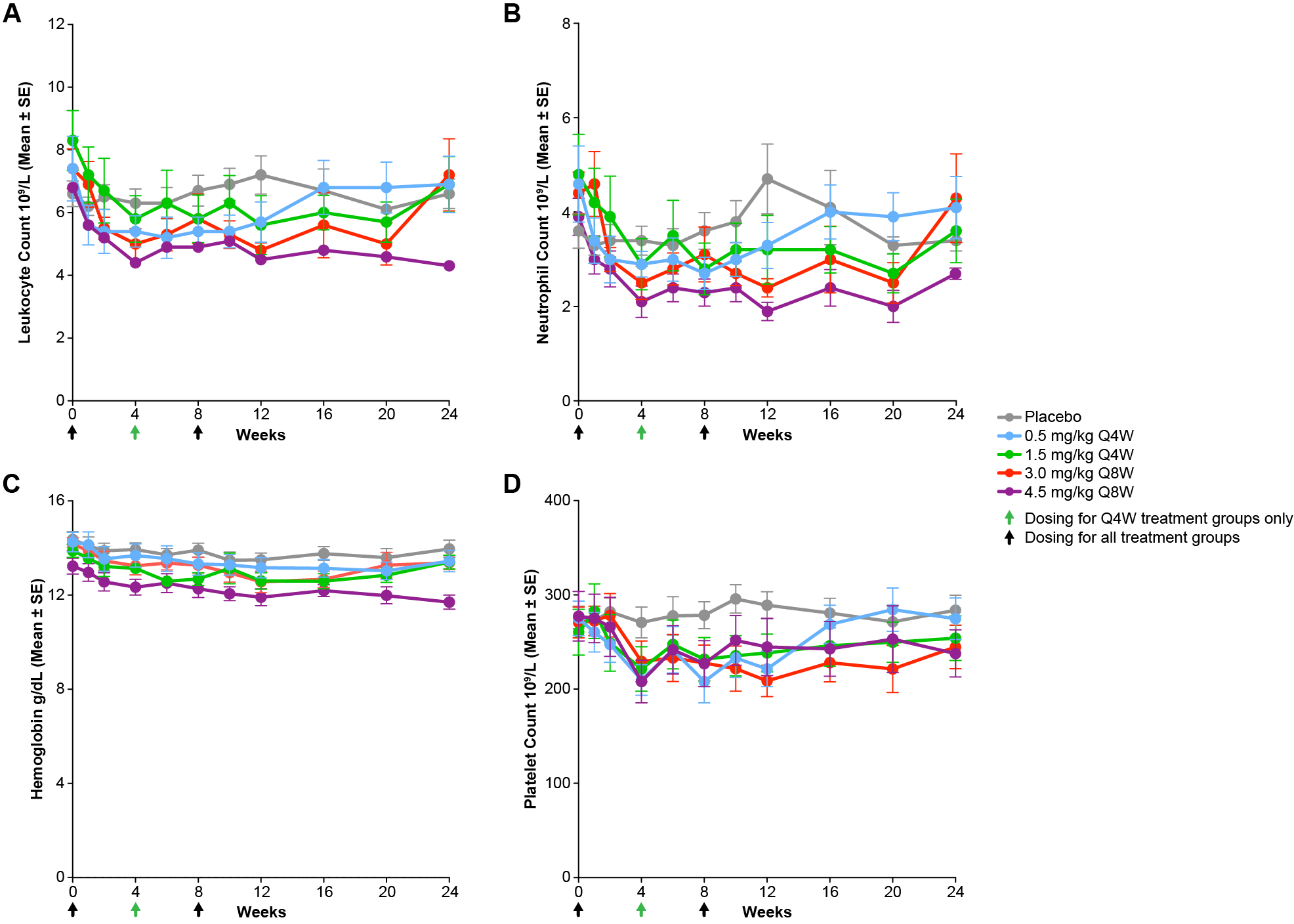


**Supplemental Figure 4.** **Change from Baseline in HSS and ISS According to Barzolvolimab Dose.** Shown is the mean score ± SE from baseline to Week 24 in each dose cohort. A) HSS7 and B) ISS7 calculated over 7 consecutive days describes disease activity, with lower scores representing low disease activity and higher scores representing high disease activity. Abbreviations: HSS, Hives Severity Score; HSS7, Hives Severity Score over 7 days; ISS, Itching Severity Score; ISS7, Itching Severity Score over 7 days; SE, standard error; Q4W, every 4 weeks; Q8W, every 8 weeks.


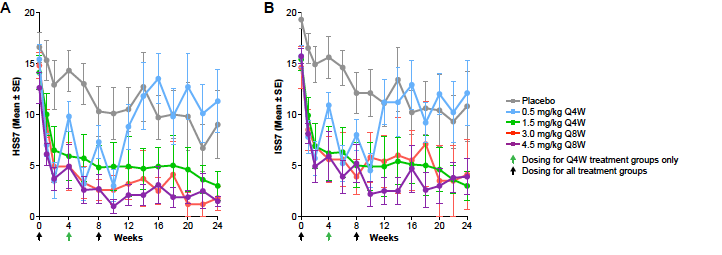


**Supplemental Figure 5.** **Change from Baseline in AAS According to Barzolvolimab Dose.** Shown is the mean score ± SE from baseline to Week 24 in each dose cohort. AAS7 describes disease activity, with lower scores representing low disease activity and higher scores representing high disease activity. *AAS7 was only calculated for patients who reported angioedema at baseline. Abbreviations: AAS, Angioedema Severity Score; AAS7, Angioedema Severity Score over 7 days; SE, standard error; Q4W, every 4 weeks; Q8W, every 8 weeks.


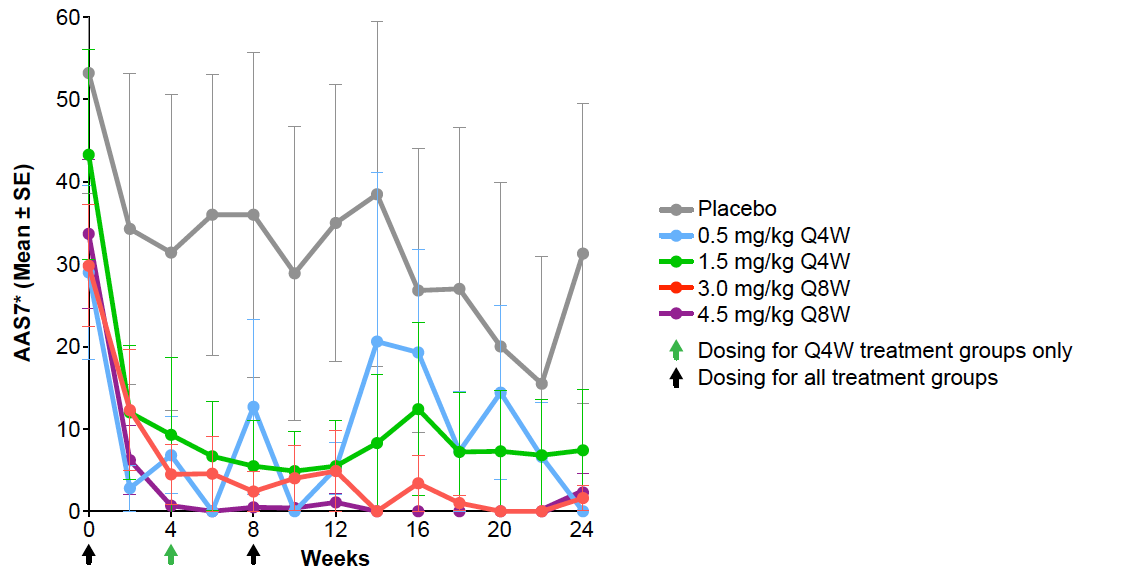


**Supplemental Figure 6. Change from Baseline in DLQI and PhysGA According to Barzolvolimab Dose.** Shown is the mean score ± SE from baseline to Week 24 in each dose cohort. A) Mean DLQI score of 0-1= no effect at all on patient’s life, 2-5= small effect, 6-10= moderate effect, 11-20= very large effect, and 21-30= extremely large effect of disease on patient’s quality of life. B) Mean PhysGA score of 0= None, 1= mild, 2=moderate, 3= severe for disease severity. Abbreviations: DLQI, Dermatology Life Quality Index; PhysGA, Physician Global Assessment; SE, standard error; Q4W, every 4 weeks; Q8W, every 8 weeks.


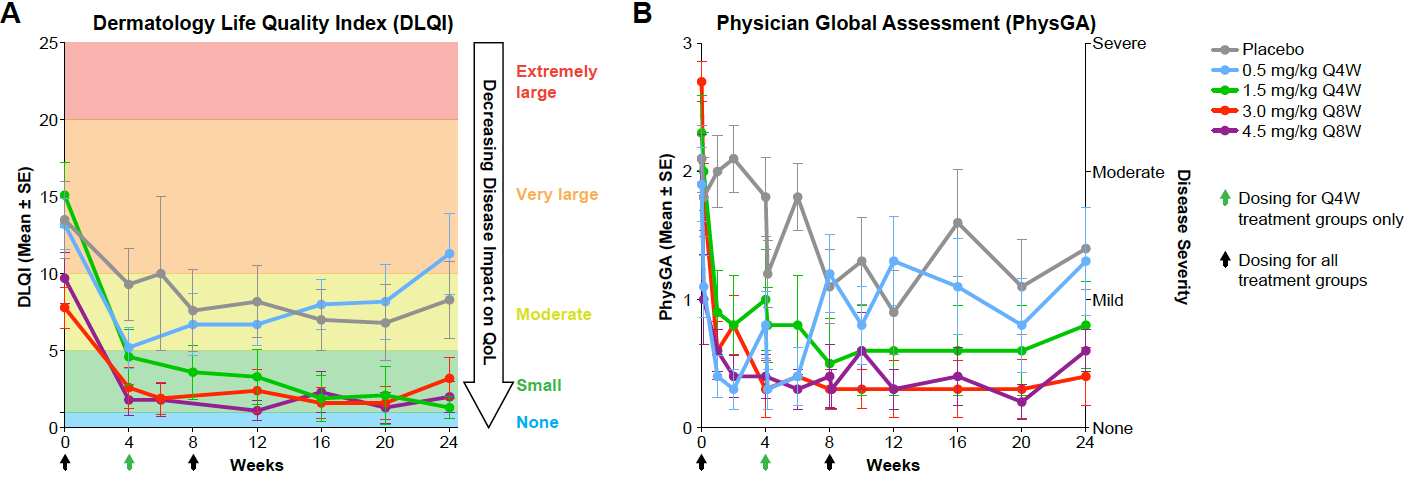


**Supplemental Table 1.** **Summary of Mean (SD) Noncompartmental Pharmacokinetic Parameters**

|  | Dose Barzolvolimab (mg/kg) | | | |
| --- | --- | --- | --- | --- |
|  | 0.5 (n=9) | 1.5 (n=8) | 3.0 (n=9) | 4.5 (n=9) |
| Cmax (μg/mL) | 15.3 (1.3) | 45.5 (9.2) | 93.0 (39.5) | 136.9 (33.5) |
| AUC_0-28_ (day*μg/mL) | 89 (15) | 367 (88) | 664 (137) | 1122 (252) |
| CL (mL/day/kg) | 5.7 (1.1)* | 3.1 (0.9) | 2.7 (0.8) | 2.4 (1.0) |
| Vz (mL/kg) | 41 (6) | 77 (31) | 94 (35) | 88 (20) |
| Half-life (days) | 5.1 (1.0) | 16.9 (3.5) | 24.8 (9.8) | 27.7 (8.7) |

*Nonlinear clearance below approximately 10 μg/mL suggests contribution from parallel mechanisms, assumedly target mediated.

Abbreviations: AUC, area under the curve; C_max_, maximum concentration; CL, clearance; SD, standard deviation; Vz, volume of distribution.
